# DNA Transfer in Forensic Science: Recent Progress towards Meeting Challenges

**DOI:** 10.3390/genes12111766

**Published:** 2021-11-07

**Authors:** Roland A. H. van Oorschot, Georgina E. Meakin, Bas Kokshoorn, Mariya Goray, Bianca Szkuta

**Affiliations:** 1 Office of the Chief Forensic Scientist, Victoria Police Forensic Services Department, Macleod, VIC 3085, Australia; 2 School of Molecular Sciences, La Trobe University, Bundoora, VIC 3086, Australia; 3 Centre for Forensic Science, University of Technology Sydney, Ultimo, NSW 2007, Australia; georgina.meakin@uts.edu.au; 4 Centre for the Forensic Sciences, Department of Security and Crime Science, University College London, London WC1H 9EZ, UK; 5 Netherlands Forensic Institute, 2497 GB The Hague, The Netherlands; b.kokshoorn@nfi.nl; 6 Faculty of Technology, Amsterdam University of Applied Sciences, 1097 DZ Amsterdam, The Netherlands; 7 College of Science and Engineering, Flinders University, Adelaide, SA 5042, Australia; mariya.goray@flinders.edu.au; 8 School of Life and Environmental Sciences, Deakin University, Geelong, VIC 3220, Australia; b.szkuta@deakin.edu.au

**Keywords:** DNA transfer, DNA persistence, DNA prevalence, DNA recovery, activity level evaluation, forensic science

## Abstract

Understanding the factors that may impact the transfer, persistence, prevalence and recovery of DNA (DNA-TPPR), and the availability of data to assign probabilities to DNA quantities and profile types being obtained given particular scenarios and circumstances, is paramount when performing, and giving guidance on, evaluations of DNA findings given activity level propositions (activity level evaluations). In late 2018 and early 2019, three major reviews were published on aspects of DNA-TPPR, with each advocating the need for further research and other actions to support the conduct of DNA-related activity level evaluations. Here, we look at how challenges are being met, primarily by providing a synopsis of DNA-TPPR-related articles published since the conduct of these reviews and briefly exploring some of the actions taken by industry stakeholders towards addressing identified gaps. Much has been carried out in recent years, and efforts continue, to meet the challenges to continually improve the capacity of forensic experts to provide the guidance sought by the judiciary with respect to the transfer of DNA.

## 1. Introduction

Awareness and understanding of the transfer, persistence, prevalence and recovery of DNA (DNA-TPPR), as well as access to relevant data on these issues to support probability assignments, have become increasingly relevant to forensic scientists to assist with investigations of alleged criminal activities and to provide guidance to the triers of fact [1,2]. The desire to utilise DNA profiling methodologies is due to the high discrimination power of the profiles they are able to generate, and thus, their ability to exculpate or inculpate an individual [3,4,5]. Further, the sensitivity of these methods allows profiles to be generated from minute quantities of biological material [6,7,8]. Knowing that profiles can be readily collected from objects that have been touched by hands [9,10] or worn [11,12,13,14] significantly broadened the scope of objects and surfaces that are targeted for DNA sampling to assist investigations [15]. Such items may include weapons, tools, containers, bags, bottles, personal items, appliances, computers, phones, tie-wraps, ropes and cords, clothing items, glasses, tape, documents, handles, steering wheels, chairs, tables and benches, windows, cartridge cases, circuit boards, stones, etc., that may be associated with any type of criminal activity including robberies, sex offences, assaults, homicides, drug manufacturing and trafficking, and a range of other indictable offences as well as items associated with missing persons cases [6,15,16,17,18,19,20,21,22,23]. Within many jurisdictions, samples collected from these types of items form a substantial portion of the samples collected for profiling and are heavily relied on to assist investigations [16,20,24].

**Figure 1:**
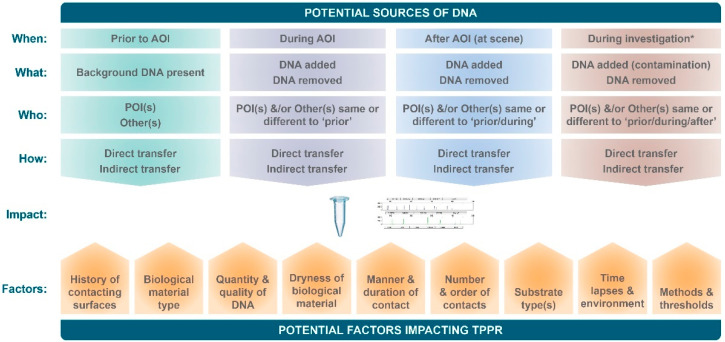
Potential sources contributing to, and factors impacting, the sample recovered and the DNA profile generated from it. The variable shading within each box represents that each source and factor can vary in relative contribution and impact. AOI = Action of Interest. POI = Person of Interest. * Apart from contamination of evidence by DNA from investigators and their associates during the investigation process, detection of AOI-related DNA could be due to cross contamination with another area of the same item or other item of the same case due to direct transfer events (for example, within packaging) or indirect transfer events (for example, via reused, uncleaned, or inadequately cleaned, tools, bench and/or gloves).

However, two elements have meant that increasingly, the question of relevance to the trier of fact has shifted from who the DNA is from, to how and when it got to the area from which it was collected [2,25,26,27]. The first is the knowledge that DNA can not only be deposited by a person contacting an object but can also be readily transferred by various other modes, including (a) from person to person to object; (b) from person to object to person to object; (c) from person to object to object [1,9]. The second is that many of the samples collected from touched objects and worn clothing [13,28,29,30], as well as samples of small stains of various biological materials such as blood, saliva and semen collected from various surfaces with pre-existing levels of background DNA [31,32,33,34], provide mixed profiles. Figure 1 depicts a general overview of the many potential sources (including who, what, when and how) that may contribute to a recovered sample and the DNA profile generated from it, as well as the many factors that could potentially impact the levels of transfer, persistence, prevalence and recovery of these contributions.

To provide guidance on the likelihood of DNA findings given competing propositions of interest with respect to the activities performed requires a thorough understanding of the factors and variables impacting DNA-TPPR, the availability of data in order to generate probabilities of particular types of profiles given specific sets of circumstances, and the knowledge of potential impact of the methodologies, processes and thresholds applied to generate the available data. In late 2018 and early 2019, three major reviews were published on elements of these, with each advocating the need for further research and other actions to support the conduct of DNA-related activity level evaluations. Firstly, van Oorschot et al. [1] conducted a review of the state of play with respect to DNA transfer in forensic science and highlighted areas requiring further attention. This was soon followed by two additional major reviews relating to DNA-TPPR: Burrill et al. [35] and Gosch and Courts [36]. 

In their review, van Oorschot et al. [1] provided a brief history of aspects relating to the transfer of DNA, indicated why understanding aspects of DNA-TPPR and availability of related data are important to the forensic and legal fraternity, plus gave a detailed summary of the many aspects pertaining to the transfer of DNA within the forensic science context. These included:

- What was known about DNA-TPPR at the time, including in relation to:
  ○ Potential means and complexities of transfer.
  ○ Core factors impacting transfer.
  ○ Prevalence and origins of non-self DNA on various surfaces.
  ○ Persistence of deposited DNA in various circumstances.
  ○ Potential relevance of activities performed between activities of interest.
  ○ Impact of using different recovery and processing methodologies on quantities of DNA retrieved and the profile types generated.
- Consideration of associated elements, including in relation to:
  ○ Contamination risks.
  ○ Type of sources of information, and tools, to utilise when addressing activity level questions.
  ○ Readiness of those addressing transfer-related questions, and the adequacies of training, competency/proficiency testing and accreditation programs.
  ○ Means of creating, gathering, sharing and using data.

The authors also highlighted several areas that could benefit from more attention/investment, including:

- Research to better understand the effect of the many variables impacting DNA-TPPR, and build the data necessary to facilitate more accurate probability assignments of generating particular types of profiles in a wide range of relevant casework situations. Specific areas of research include:
  ○ Impacts of physical and chemical differences of contacting surfaces, including those relating to their topography, chemical compositions, fibre type, weave, thickness, electrical charge, etc.
  ○ Assessment of transfer of primary touch deposits after long time periods.
  ○ Drying patterns and transfer rates of various relevant biological materials, such as semen, and the impact different substrates may have.
  ○ Effect of duration and/or frequency of use on the accumulation of DNA, and subsequent changes to profiles, for objects of different types and substrates.
  ○ Awareness of general levels of background DNA (including quantity, origin and quality), and the impact of factors such as a person’s shedder status and frequency of item use.
  ○ Understanding of the influence of any background DNA on the interpretation of mixed profiles.
  ○ Acquisition of more accurate probabilities of finding non-self DNA, and of the different relative mixture proportions within a deposit made by a hand, by collecting more samples from random individuals in a wide range of situations.
  ○ Prevalence of non-self DNA on the bodies of both children and adults.
  ○ The quantity and quality of DNA from donors and other individuals in fingernail samples after various known contact activities; acquisition of non-self DNA during regular social interaction; indirect transfer by occupying another person’s space for a certain amount of time; the effects of personal habits; and an individual’s shedder status, on detection of donor and foreign DNA.
  ○ Awareness of contributors to the non-self component of DNA retrieved from personal objects and occupied spaces.
  ○ Persistence of the temporary user of an item after use by the original owner has resumed.
  ○ Probabilities of detection, and relative contribution, of individuals to profiles retrieved from a wider array of shared objects and surfaces within confined shared spaces (e.g., homes, offices, cars) and public spaces, given known histories.
  ○ Impact of handwashing on the amount and quality of DNA deposited from hands, including factors such as: different methods of washing hands, the natural accumulation of DNA on hands post-handwashing, and the different personal habits of individuals, and their effect on the accumulation of self and non-self DNA on hands.
  ○ Impacts of genetic factors and various non-genetic factors (e.g., behavioural traits, health situation, and/or environmental conditions), and their potential interactions, on shedder status, as well as identifying and understanding their underlying properties.
  ○ Understanding the impact of the absence/presence of the types of information one relies on that may be gathered by the crime scene attending officer, and to consider means of improving the gathering of relevant information in an efficient and consistent manner.
  ○ Studies to help establish more accurate probability distributions for relevant factors impacting transfer.
  ○ Determining the accuracy of ‘experiential’ assessment (i.e., based on experiences) relative to ‘experimental’ assessment (i.e., based on data from experiments).
- Enhancement of access to relevant data, including specific areas such as:
  ○ Availability of a quality controlled open access depository of relevant DNA-TPPR information that can be easily mined for various purposes.
  ○ Inclusion of relevant details of the methods, protocols and thresholds applied to generate the DNA-TPPR data presented.
- Recognition of DNA activity-associated expertise to be distinct and supported by dedicated training, competency testing, authorisation and ongoing proficiency testing.

In the review by Gosch and Courts [36], an excellent summary of the existing literature as of October 2018 was also provided with respect to our knowledge of the core variables influencing DNA-TPPR. They highlighted the limitations of much of the DNA transfer-related research studies conducted to date in terms of:

- Underestimating the complexities and multitude of factors needing to be considered.
- Recording relevant information to allow comparisons and appropriate use.
- Identifying the relative weight of different impacting variables.

Regarding the latter, they pointed out that factors such as casework-relevant persistence scenarios or the impact of inter-individual differences in handling an item are among those receiving limited attention. The authors further highlighted deficiencies in overall research study designs and documentation, lamented on the lack of agreement on the DNA analysis output parameters most appropriate to be utilised when comparing studies and addressing activity level questions and standardisation of recording these, and indicated a disequilibrium in the degree of studies relating to pertinent factors. Furthermore, they described and launched a new database (‘DNA-TrAC’) [37] to assist the accessibility of relevant DNA-TPPR information to aid addressing of activity level questions.

Around the same period, Burrill et al. [35] also provided a review of published research on aspects of transfer and persistence relating to ‘touch DNA’, including on:

- How much DNA can be recovered from handled items.
- Whether trace DNA can be detected under certain scenarios including varying degrees of indirect transfer.
- Factors which may influence these results.

Whilst the authors pointed to similar issues raised by the two above mentioned reviews, they also explored deeper the current knowledge in relation to the composition of touch deposits. They concluded that ”Further exploration of relative contributions of hand-endogenous and -exogenous DNA contributions to touch deposits is warranted, along with more specific localization of DNA sources inside and outside a cellular infrastructure”, and that “A more comprehensive picture of where the DNA in ‘touch DNA’ comes from will provide valuable information to practitioners seeking to utilize touch DNA typing results in a courtroom context, and will contribute to the ongoing effort to understand and predict the circumstances of DNA transfer in a forensic context more reliably”.

A year prior to the three reviews mentioned above, in their Appendix B, Taylor et al. [38] identified 17 study areas that, if acted on, would improve our knowledge base and assist evaluations of evidence in light of propositions that suggest differing DNA transfer mechanisms. These specific studies related to areas including: efficiencies of different extraction methods, substrate considerations, environmental conditions, persistence of DNA in various situations, shedder consistency and shedder propensity determination, duration and manner of handling/contact, objects used habitually, accumulation of DNA from multiple contacts, levels of primary user’s DNA on regularly used items, levels of background DNA, and value of profile degradation information.

Collectively, the aforementioned three reviews [1,35,36] and the research paper by Taylor et al. [38] acknowledged the importance of DNA-TPPR in investigations of criminal activity, highlighted the many limitations in our current knowledge and availability of required data, and provided some guidance on improvement opportunities. In this paper, we assess the extent to which we are meeting these challenges. This is achieved primarily by providing a synopsis of publications relevant to DNA-TPPR that have been published since these reviews. We then reflect on this and some of the actions taken by industry stakeholders in support of addressing identified gaps. Finally, we provide a general conclusion in relation to progress towards improving the guidance that experts are able to provide the courts in relation to the transfer of DNA.

## 2. Identification and Reporting of Recent DNA-TPPR Publications

Identification of recent relevant publications was achieved through the conduct of a systematic review. The search was conducted on 30 March 2021 for the period 2018 to the present within Scopus and Web of Science. Applying the Preferred Reporting Items for Systematic Reviews and Meta-Analyses (PRISMA) guidelines [39]. The search commenced by applying the following:

TITLE-ABS-KEY ((DNA OR “deoxyribonucleic acid”) AND (transfer* OR deposit* OR contact* OR persist* OR prevalen* OR background OR shedder) AND (human OR participant OR donor OR volunteer) AND (forensic* OR crim*)) AND (LIMIT-TO (PUBYEAR, 2021) OR LIMIT-TO (PUBYEAR, 2020) OR LIMIT-TO (PUBYEAR, 2019) OR LIMIT-TO (PUBYEAR, 2018)).

**Figure 2:**
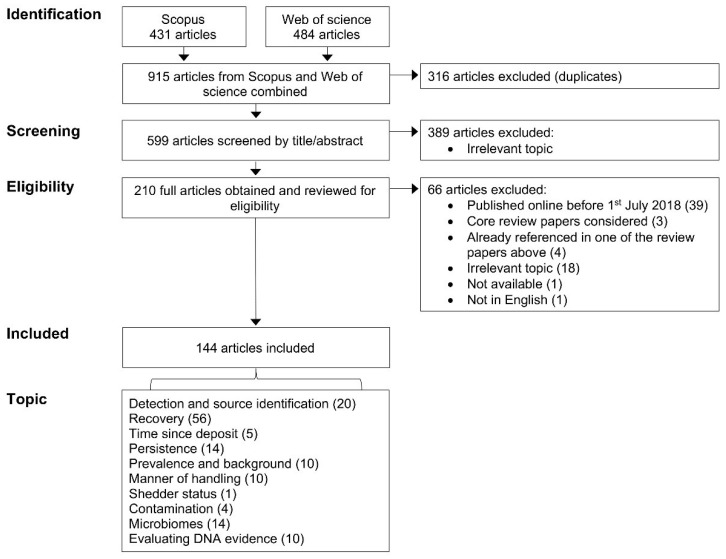
Process and outcome of review to identify articles to be included in synopsis. Topic categorisation of articles was based on their primary area of DNA-TPPR-related focus. Numbers of articles included/excluded are indicated in parentheses after each topic/exclusion criterion.

One hundred and forty-four papers were identified for inclusion in the synopsis of relevant publications within the given period presented in Section 3 (Figure 2). As we may not have captured some relevant publications, or inadvertently excluded some from further consideration, we advise those seeking publications relating to specific core topics to conduct additional searches relating to their narrower area of focus.

Based on the primary DNA-TPPR-related focus and content of the 144 included articles, each was assigned to one of the following topic categories: detection and source identification, recovery, time since deposit, persistence, prevalence and background, manner of handling, shedder status, contamination, microbiomes, evaluating DNA evidence. Where a paper was deemed to also contain information of relevance to an additional topic area, this information was also conveyed within the other topic subsection.

A synopsis is provided that gives a general overview of the focus areas and some general findings of the articles reviewed. The articles included in the synopsis were from many different journals, with *Forensic Science International: Genetics* (54) and *Forensic Science International* (23) providing the most articles. The majority of papers focused primarily on various aspects of DNA detection and recovery, with fewer concentrating on various elements of transfer, prevalence and persistence (Figure 2). The degrees of relevance of the publications identified vary, yet each is incorporated and referred to in the synopsis. The scope and depth of some of the presented publications are very limited. Further, some of the experimental designs within the studies conducted have limitations and while some acknowledge this, others do not. However, we have refrained from drawing attention to these and, instead, urge readers to delve fuller into the papers of interest to make their own assessment of such matters.

The reported findings of conducted research are those determined by the authors of the research and not our extrapolations. While in general we concur and/or are supportive of the vast majority of findings, conclusions and recommendations reported in the publications listed, their inclusion does not necessarily imply that we concur, and we do not make systematic comparisons of studies to inform a further judgement. For more details of the experimental designs, methodologies utilised, analyses employed, results obtained, and associated interpretations and discussion of the studies referred to, we recommend that the relevant specified references are consulted.

Several of the reported findings are new and broaden our understanding and/or improve the availability of relevant data and some produce findings verifying existing understandings/knowledge with or without making reference to relevant prior studies. We have chosen not to point out connections to prior studies here, but urge interested parties to consult the details of the publication of interest as well as any others on the relevant topic that were published prior to the period of publications bookending in this review to gain a fuller appreciation of the available knowledge and data. References within the papers of interest listed here and those listed within the aforementioned review articles can be a good start in such situations. Further, we are aware of some interesting and relevant papers that have been published since the cut-off date for inclusion within this review, and thus, are not included here. A selection of these more recent articles will be highlighted in the discussion section.

## 3. Synopsis of Recent DNA-TPPR-Related Publications

### 3.1. Detection and Source Identification

Twenty papers were identified that primarily addressed detection of biological sources and/or their identification (Figure 2), plus one additional study that also addressed this issue but was primarily focussed on another topic. Several studies investigated the visualisation and detection of biological materials. In addition to readily visualising biological stains, Yano et al. [40] demonstrated the ability of an acid-free *p*-dimethylaminocinnamaldehyde solution sprayed on glass slides and clothing and irradiated with blue-light-emitting diode light to visualise relatively fresh deposited finger and palm prints on clothing. This study also demonstrated that this technique does not adversely affect STR analyses, thus providing potentially useful methodology to localise areas on items of interest to assist better targeting for sampling. A number of other studies also demonstrated the ability of various treatments to potentially enhance detection of biological materials, including the use of:

- Trypan blue to improve visibility of shed skin flakes collected by adhesive film [41].
- Diamond™ Nucleic Acid Dye (DD) (Promega, Madison, WI, USA) to detect cellular material in lip-prints [42].
- DD to detect DNA on used swabs to potentially assess if further processing for DNA profiling is warranted [43].
- Ethidium Bromide to detect small amounts of DNA on swabs to possibly provide similar pre-screening benefits [44].
- Immunohistochemical staining of skin-expressed proteins to identify exfoliated epidermal cells to screen samples taken from handled/touched objects [45].
- NIR hyperspectral imaging to localise invisible traces of blood, urine and semen on various substrates [46].
- A test based on the click reaction between serum albumin and tetraphenylethene maleimide (TPE-MI) to visualise invisible blood stains (and showing that it may perform better than luminol) [47].
- Fluorescence spectroscopy to localise the presence of saliva stains [48] or detection of saliva on swab samples of human skin that had been licked [49].

Furthermore, Butler et al. [50] compared four methods that have been previously used as presumptive tests for the detection of traces of blood on dark materials, finding that luminol was, overall, the most effective method.

To explore source differences and gain insight into associations with DNA content, Burrill et al. [51] used flow cytometry and microscopic examination to generate granularity, size and nucleic acid fluorescence data of washed and unwashed hands, as well as saliva, nasal and eye wash that could be sources of transferred DNA onto hands. They found that hand rinses consisted mainly of anucleate corneocytes, many of which also stained positive for nucleic acids, and suggested the need for further research on the recovery and analysis of corneocyte DNA, which they report was previously assumed to be negligible. 

The potential of a software tool for the automated detection and characterisation of epithelial cells from fingerprints based on fluorescence intensity across the cell surface and size/morphology was demonstrated by Olsen et al. [52]. This study also observed that trace cell populations from certain individuals may have distinct morphological and/or structural properties. The authors pointed out that this technique can, therefore, facilitate investigations into the deposit, transfer, and persistence of both cellular material and DNA in trace biological samples.

Tests for the detection of human PSA and human α-amylase for the identification of seminal fluid and saliva, respectively, in forensic samples are commonly used as presumptive tests in sexual assault-related cases to aid decisions on further testing procedures. It is relevant to know how frequent PSA and amylase are detectable by these tests in vaginal swabs that are void of seminal fluid and saliva. A study by Kishbaugh et al. [53] of 50 vaginal swabs from unique donors over the age of 18 years and relevant history, and a study by Sari et al. [54] of 20 pre-pubescent (3–9 years), 20 post-pubescent (16–45 years) and 20 post-menopausal (50–75 years) females, help fill this knowledge gap, and thus, assist the accuracy of interpretation of immunochromatographic testing results. In addition, a review of current methods of sperm detection, factors affecting sperm detection, and recent advances in sperm detection utilising alternative methodologies is provided by Suttipasit [55].

Traditional identification methods for the nature of the body fluids or tissues present in a sample collectively cater for a narrow range of biological materials tend to require one test per target fluid or tissue and may consume material compromising attaining a DNA profile. New methodologies, especially those utilising mRNA and proteomics approaches, have shown opportunities to identify a wider range of biological materials that may enhance forensic examination capacities. Molecular biology techniques continue to be explored to enhance the capacity to improve source identification. Abbas et al. [56] reported on the development of a protein microarray chip with enhanced fluorescence for sensitive identification of semen and vaginal fluid and pointed to the value of further development of highly sensitive chips for the identification of other biological body fluids. O’Leary and Glynn [57] highlighted the potential of a selection of miRNAs to identify forensically relevant body fluids. Hanson et al. [58], using cSNPs in body fluid-specific mRNA transcripts, demonstrated the ability to identify forensically relevant body fluids and skin, as well as differentiate blood, semen and saliva transcripts from different individuals. Those authors also indicated that, with further development, one may in the future be able to assign body fluids to DNA donors, which, in many instances, may provide additional probative value. Kamanna et al. [59] demonstrated the potential of proteomic analysis, using mass spectrometry-based approaches, MALDI-ToF MS/MS and n-LC-ESI-qToF MS/MS, to identify vaginal fluid and blood from only a few fibres plucked from a microswab used to collect a sample from underneath fingernails. In addition, Watanabe et al. [60] explored the simultaneous analyses of semen-specific methylated (or unmethylated) CpG markers and adjacent single nucleotide polymorphisms. By combining methylation-specific polymerase chain reaction and pyrosequencing technology, the authors developed a method to detect three semen-specific methylated/unmethylated regions that was able to identify semen-derived alleles from mixed stains. The authors suggested that this type of methodology may be a useful approach for genotyping from a mixture of body fluids.

### 3.2. Recovery

This section includes studies that investigated various aspects of DNA recovery, specifically sampling, storage, extraction, preferential recovery from mixed samples, profiling, and differences in the methodologies employed. Fifty-six papers are reported (Figure 2), along with some others that were primarily included in other topics.

#### 3.2.1. Sampling

Several studies continue to elucidate the ability to generate DNA profiles from samples taken from touched objects [61,62,63,64]. Additional studies evaluated means of generating human DNA profiles from traces of biological material left on various items, including hair [65], bricks [66], dogs [67], bite marks in food [68], paper documents [69], eye wear [70], superabsorbent polymer-containing products (commonly found in nappies and sanitary towels) [71], improvised explosive devices [72], rifle magazines [73] and bullet cartridges [74,75,76,77,78]. Further, Gray et al. [79] demonstrated that STR DNA profiles can be generated from human blood found in *Anopheles* mosquitoes, from one or multiple individuals, and that the quality of the profiles declined as the duration between mosquito blood meal and sample collection increased. 

The performance of different swabs on the collection and profiling of DNA continues to be explored. When considering touch DNA recovery, two studies compared the performance of different swabs, with each finding that a specific type of swab performed better than others [80,81]. A further study indicated that the use of pieces of absorbing paper moistened with 70% ethanol recovered epithelial cells/touch DNA from various surface types better than a cotton swab moistened with water [82]. In addition, Sherier et al. [83] illustrated the potential benefits of using the microFLOQ^®^ Direct Swab (Copan, Brescia, Italy) for collecting DNA from minute stains, and possibly using it as a pre-screening methodology (i.e., sampling a small portion of a stain for initial screening and testing for expeditious processing) while saving the remainder of the stain for later testing. They also demonstrated that swabbing the centre of stains of blood, semen and saliva provided more DNA and complete profiles than swabbing the edge of stains.

Investigation also continues into the use of adhesives, such as tapes and gels, for DNA sampling. Kanokwongnuwut et al. [84] compared the efficiencies of different types of tape on collecting DNA from fingermarks and the effect of different numbers of applications, illustrating that particular tapes performed better than others and the benefits of ten applications compared to just one or two applications. Damsteeg-van Berkel et al. [85] showed that collection efficiency from textile substrates increased with increasing stubbing force in a concave down increasing function to a threshold depending on the substrate material. Zorbo and Jeuniaux [86] illustrated that DNA profiles can also be generated from cells, particularly those deposited by skin contact, collected using 1:1 tapings that are conventionally used to obtain microtraces of fibres and hairs prior to sampling for DNA from a body or clothing. However, the authors recommended taking additional samples from an area of interest post 1:1 taping using other techniques such as swabbing. Dierig et al. [41] compared DNA yields and profiles of single skin cells recovered from adhesive tape versus processing of 1 × 1 cm areas of adhesive tape after the adhesive tape had been applied to external areas of T-shirts after a period of wearing and where they had been vigorously grappled for 10 sec to mimic a physical assault. They found the approach of sampling 1 × 1 cm areas to be superior in regard to DNA yield, profile completeness and identification of the offender profile. Finally, gels have also been shown to be efficient methods for the recovery of DNA from fingermarks [87,88,89,90].

A few studies point to the potential benefits of applying different collection methods depending on the type of substrate [91], especially with respect to metal surfaces [80,92]. In addition, Sessa et al. [93] compared three sampling techniques (adhesive tape, cutting out, dry swab) for the detection of touch DNA from wearers and handlers of brassieres, and found no difference in the recovery of the handler’s DNA among the three techniques, even though the cutting out technique showed the greatest recovery. However, recovery of the wearer’s DNA was best achieved using adhesive tape. Overall, for their sets of samples, use of the dry swab collection method performed worst. However, for the subset of samples where in the experimental design the period of contact was shortest, out of the three techniques, samples collected using the dry swab technique yielded the highest number of handler profiles. The authors also demonstrated that when targeting for foreign DNA, the sample area should be narrowed as much as practicable to the smallest area possible to maximise target DNA recovery.

In addition to its use in visualising areas of cellular deposits, DD has also been used to determine how many cells are required to generate an informative DNA profile, either by direct PCR or conventional DNA profiling methodologies [94]. This study found that, when using direct PCR, ≥40 buccal cells collected from a saliva sample by either a swab or tapelift were required, whereas, from a touch sample, ≥800 corneocytes collected by swabbing or ≥4000 corneocytes collected by tapelift were required. However, more cells were required for the same DNA profile result when samples were processed through a DNA extraction workflow: ≥80 buccal cells from a swab and tapelift, ≥4000 corneocytes collected by a swab, ≥8000 corneocytes collected by a tapelift, illustrating that the use of direct PCR can be more efficient. The authors also concluded that cell-free DNA forms a major component of the DNA collected from touched deposits, and that tapelifting is not as efficient as swabbing in co-collecting this component of the total DNA deposited. Miller et al. [95] and Burrill et al. [96] also provided evidence that extracellular DNA can represent a substantial proportion of total DNA recovered from handled substrates. Additionally, Burrill et al. [96] determined a suitable technique to separate cellular and cell-free fractions of hand deposits (i.e., one that maintains the representative fraction of cell-free DNA at the time of collection) and the best of three methods tested to purify the cell-free DNA for DNA profiling. A separate study by Burrill et al. [97] demonstrated that enhanced lysis methods using a reducing agent and longer incubation may be valuable for the extraction of DNA from corneocytes, which make up the majority of the cellular material left in touch deposits by hands. The authors also showed that the corneocyte DNA is fragmented. The authors suggested that use of methods that extract the DNA from corneocytes more efficiently than standard methodologies, and application of profiling techniques more suitable to fragmented DNA, could enhance the ability to obtain useful profiles from touch DNA samples.

#### 3.2.2. Storage

Three studies investigated the impact of storage conditions on the preservation of DNA in biological materials. Kaur et al. [98] looked at the degradation (using UV–visible spectroscopic profiles) of various types of forensic blood-related casework samples preserved under various conditions and different durations. They found that the preservation techniques had a greater impact on the quality of DNA than the aging of the sample, but not to the degree that STR profiles could not be generated. Badu-Boateng et al. [99] examined various storage conditions to determine the most appropriate means of storing crime scene soil–blood mixed samples prior to analysis at the laboratory, based on DNA quantification, DNA degradation and DNA profile parameters. They found that storage at −20 °C was the best for this sample type, followed by room temperature and 4 °C, respectively. Corradini et al. [100] showed that buccal cells collected directly on to FTA mini cards (GE Healthcare) and stored for 11 years still provided good quantities of DNA and good-quality DNA profiles. In addition to these studies, Suttipasit [55] emphasised the need for appropriate long-term specimen preservation in order to be able to take advantage of potential future methodological advances to interrogate samples beyond current abilities.

#### 3.2.3. Extraction

Several studies reported the benefits of using direct PCR methods over processes that include a magnetic bead extraction step for the generation of DNA profiles from touched objects, including Kanokwongnuwut et al. [94]. Dierig et al. [41], comparing different DNA extraction methods, found that direct lysis methods (a Chelex solution method, Casework Direct Kit (Promega, Mannheim, Germany), Investigator Casework GO! Kit (Qiagen, Hilden, Germany)) were more suitable than a magnetic bead-based method (Maxwell RSC Blood DNA Kit (Promega)) for low template traces due to the limited loss of DNA. Meanwhile, de Oliveira Francisco et al. [101] found that the Casework Direct Kit (Promega) was much more efficient for processing touch DNA samples than the DNA IQ™ System (Promega), another magnetic bead-based DNA extraction method.

A couple of studies looked toward the future, considering how we might better understand and improve extraction efficiencies. LeSassier et al. [102] described means of developing artificial fingerprint samples and advocated their use to assist future studies on the efficiencies of various methods of collection and extraction of DNA from fingerprints. Liu et al. [103] summarised the recent development of magnetic solid-phase extraction in forensic science applications, including DNA extraction, and pointed out that modifications of functional materials on the surface of magnetic particles that could lead to potential improvements (e.g., to better cope with negative impacts of some pre-processing tools, such as fingerprint powder and tapes) and broadening of their application (e.g., detection of biomarkers, such as amino acids, peptides, hormones, etc.).

Two studies considered the extraction of non-DNA elements of biological samples for forensic applications. O’Leary and Glynn [57] identified the best of three methods investigated for the extraction of miRNAs from body fluids for the purpose of using miRNAs for body fluid identification. A study to support the potential future application of genetically variable peptide analysis of touched samples for forensic identification [104] considered a workflow for the collection, enrichment and fractionation of DNA and protein in latent fingerprint samples. The authors demonstrated that the workflow provided sufficient DNA and protein from touch samples for further analysis. Further, the authors observed that the quantity and quality of protein remained robust, regardless of fingerprint age, and that the proteomic content of the touch samples was consistent across donors and fingerprint age.

#### 3.2.4. Preferential Recovery from Mixed Samples

Some studies considered the impact of different sampling and pre-sampling methods on preferential recovery of DNA from particular donor(s) in mixed samples. Hayden and Wallin [105], investigating the effectiveness of different methods of sampling from fingernails to minimise the collected amount of endogenous (female) DNA and maximise the amount of exogenous (male) DNA, concluded that their swabbing method did this better than two alternative methods tested. One study compared an automated procedure to a manual procedure for standard differential extraction of sperm-cell fractions, and found that similar levels of semen separation efficiency were achieved for sperm-cell fractions using both automated and manual procedures [106]. Three studies continue to look at novel means of separating sperm cells from other cells to assist the interpretation of DNA profiles generated downstream. These include the application of optical tweezers [107], acoustic differential extraction technologies [108], and capillary zone electrophoresis [109]. Furthermore, using DEPArray™ technology (Menarini Silicon Biosystems, Bologna, Italy), Anslinger et al. [110] demonstrated the ability to obtain complete DNA profiles from isolated single white cells from each of the two or three known contributors to mixed stains. They concluded that this technology opens up new possibilities with respect to mixture deconvolution, especially for mixtures composed of cells of the same type. However, as touch samples can contain a substantial proportion of extracellular DNA, Miller et al. [95] pointed out potential challenges for front-end cell sorting approaches to recovering DNA from touch samples, which necessitate the presence of cells with sufficient amounts of intracellular DNA.

#### 3.2.5. Profiling

Two studies demonstrated improvements in the abilities to generate Y-STR profiles from non-semen sexual assault samples. Henry and Scandrett [111] showed that the Yfiler^®^ Plus kit (Thermo Fisher Scientific, Melbourne, Australia) significantly outperformed AmpFlSTR Yfiler^®^ (Thermo Fisher Scientific) in the ability to generate useful profiles from semen negative sexual assault samples, i.e., swabs that gave negative results to a screening test for acid phosphatase or ABAcard^®^ p30 antigen test (Abacus Diagnostics, West Hills, CA, USA) in combination with the absence of spermatozoa by microscopy, and excluding samples that tested positive for the presence of human salivary amylase using the RSID™ Saliva test (Independent Forensics, Lombard, IL, USA). In contrast, Owers et al. [112] showed that Powerplex^®^ Y23 (Promega, Madison, WI, USA) significantly outperformed Yfiler (Thermo Fisher Scientific, Waltham, MA, USA) in the quality of profiles generated from vaginal swabs of alleged penetration where no semen was detected.

Two studies also investigated the use of non-traditional DNA profiling systems, specifically field-based systems and Massive Parallel Sequencing (MPS). While rapid field-based DNA identification systems have their benefits, Dawnay et al. [113] illustrated the limitations of the ParaDNA Field Instrument and Intelligence Test Chemistry (LGC) to collect viable DNA samples and produce profiles from the surfaces of the skin, exposed tissue or carrion larvae. The use of MPS in comparison to traditional Capillary Electrophoresis (CE) techniques to generate DNA profiles from real casework samples obtained from semen, saliva, blood, and epithelial material on various surfaces, as well as reference oral swabs, was evaluated by Avila et al. [114]. They found that the biological nature of the samples impacted various relevant MPS sequencing, and base-calling quality metrics associated with producing a DNA profile, not apparent when generating DNA profiles using CE; this was especially so for epithelial samples. The authors advocated for further research on MPS genotyping of forensic casework samples, including evaluation of DNA transfer effects, impact of the substrate or deposition surface for each sample and other aspects of the nature of individual samples.

Interpretation of DNA profiles and mixture deconvolution requires use of software and laboratory-specific interpretation methods. Mortera [115] reviewed the different methods and software available to assist in determining whether or not the DNA of a given individual is present in a sample. The author provided details on a quantitative method that used Bayesian Networks as a computational device for efficiently computing likelihoods. This method also allowed the combination of evidence from multiple samples to make inferences about relationships from DNA mixtures and other more complex scenarios [115]. Furthermore, Buckleton et al. [116] revisited a previously conducted interlaboratory exercise (comprising five mock cases, with each case consisting of an electropherogram file and typed reference profiles from individuals described as victims, consensual partners, or suspects) (NIST MIX13) to re-evaluate DNA mixture interpretation using currently available methods, especially several modern probabilistic genotyping systems. Their analyses demonstrated the benefits of utilising ‘probabilistic genotyping’ over ‘combined probability of inclusion’ methodology.

#### 3.2.6. Methodology Differences

In the studies by Szkuta et al. [117] and Szkuta et al. [118] of DNA quantities and profiles generated from samples collected from clothing, and by Goray et al. [119] of DNA quantities and profiles generated from samples collected from items/surfaces within office spaces, similar sets of samples were processed by different laboratories using different suites of processing, from sample collection through to the generation and analyses of DNA. These studies showed that differences in methodologies applied between the laboratories appeared to impact the quantity of DNA recovered and the composition of the profiles produced. When determining probabilities using data from various studies, Gill et al. [120] stressed the need to consider the differences in methodologies used by different studies to generate the available data.

### 3.3. Time since Deposition

Five studies were identified that considered how one might determine time since deposition (TSD) of biological materials (Figure 2), specifically blood, semen and saliva. Weber and Lednev [121] provided an overview of what is known about the biochemical mechanisms of blood aging, how these are used to determine the age of a bloodstain, the current techniques for bloodstain age determination, emerging spectroscopic techniques and technology, and the limitations of all these methodologies. Stotesbury et al. [122] and Cossette et al. [123] presented further research on using visible absorbance spectroscopy and DNA degradation to explore their use in determining TSD of blood, with the latter publication focusing on the impact of temperature. Bird et al. [124] considered degradation patterns of semen-specific mRNA genes to assist with determining TSD of semen. Meanwhile, Asaghiar and Williams [125] evaluated the use of changes in the expression of a hypoxia-sensitive marker for determining the age of deposited samples of blood, semen and saliva. Each of these studies demonstrated scope for potential use of the methodologies they investigated in TSD determination. They also noted the limitations in the scope of sample types analysed and the accuracy of TSD analyses, and recommended further research. Additionally, Weber and Ladnev [121] pointed out the need of any methodology being considered for use in forensic casework to be validated and that this would require further research into the impacts of environmental and substrate effects.

### 3.4. Persistence

This section summarises the fourteen papers identified that investigated the persistence of DNA from various biological materials (Figure 2), plus one additional study that also provided comment on persistence but was primarily categorised in another topic. Whilst it is possible to generate DNA STR profiles from a 100 year old semen stain [126] and from fingerprints archived for several years [127], when considering persistence of DNA in fingermarks left at room temperature for up to just one week, Alketbi and Goodwin [128] indicated that the quantity of DNA recovered was not influenced by the duration that the fingermarks were left before sampling, but was influenced by the environmental conditions (temperature and humidity) and type of substrate on which they were deposited.

When investigating the persistence of DNA from blood and keratinocytes deposited on a variety of porous and non-porous substrates, as well as a range of mock exhibits with natural deposits of touch DNA and saliva, under various environmental conditions (indoor temperature controlled, indoors ambient temperature, outdoors forest area in Singapore where rainfall is abundant and relative humidity is high) for a duration up to 85 weeks, Lee et al. [129] found that degradation and persistence of DNA are highly dependent on the environment. DNA on articles left outdoors degraded rapidly, but most samples collected from the items kept within indoor environments were less affected and provided good profiles for long periods after deposition, similar to trends observed in studies performed in temperate countries.

Several studies investigated the persistence of DNA on surfaces of the body (skin, beneath fingernails and hands) when immersed or washed in water under varying conditions. Meixner et al. [130] found that DNA profiles can still be generated from blood stains and ‘touch’ deposits on skin specimens after several days of being immersed in water (cold, room-temperature or warm water as well as in a stream and a pond). The duration of persistence was dependent on the temperature and aquatic environment and was longest when immersed in cold water. Hayden and Wallin [105] demonstrated that DNA can remain under fingernails (female fingernail donor’s consensual partner) and on the skin (male sweat donor’s consensual partner) after showering. While Romero-García et al. [131] noted that samples taken from towels used once to dry hands immediately after having washed them with neutral pH soap only provided partial profiles of the person drying their hands. This was the case both after the individual’s hand performed various daily activities and in separate tests where individuals had held hands with someone else for 5 min prior to washing their hands.

Other studies looked at the persistence of DNA and biological fluids on various non-porous and porous items after cleaning or washing. Helmus et al. [132] investigated whether DNA traces (blood, saliva, epithelial cells) on different objects (knives, plates, glasses, and plastic lids) can persist on the surface despite cleaning by different methods (such as washing by hand or use of a dishwasher). They found that small deposits of biological material often still provided sufficient DNA to generate full profiles after the item they were on was rinsed or washed by hand, while profiles were not able to be generated after cleaning in a dishwasher. Further studies showed that sufficient DNA for STR profiling could be recovered from cuttings of semen stains on polyester and/or cotton fabric after washing, with the amount of DNA dependent on the type of washing applied [133,134,135]. Noël et al. [133] showed that this was possible even after multiple washings, and that more DNA was recovered from cuttings than swabbing of stained cotton bedsheets. Both Noël et al. [133] and Karadayi et al. [134] also showed that the washed stains were able to be visually detected using a light source and provided positive prostate-specific antigen test results. Similarly, Hofmann et al. [136] showed that washed blood stains can generally also be detected using luminol, but the quality of the bloodstains is dependent on a number of factors including the type of washing machine, the washing temperature, the filling degree of the washing machine, and the drying conditions of the blood stain (temperature, duration).

To provide a dataset that may help guide sample targeting and prioritisation, as well as manage expectations of stakeholders regarding observing positive or negative results, Fonneløp et al. [137] performed a retrospective study on the transfer, persistence and recovery of sperm (from internal vaginal swabs, external vaginal swabs, rectal swabs, oral swabs and skin surfaces) and epithelial cells (from external genital swabs, hand swabs, skin swabs, penile swabs, internal vaginal swabs, rectal swabs and oral swabs) collected in sexual assault casework. The samples collected from different areas of the skin to assess the presence of the person of interest (POI)-derived epithelial DNA included face, lips and around the mouth, neck/throat, breast/chest, arm, leg, seat, thigh, rest of body. The authors found that sperm cells had the highest persistence rate in internal vaginal swabs, and were detected up to 72 h post assault, but the majority of the positive samples (i.e., case specific POI DNA profile detected) were collected within 48 h. Analysis of skin and penile swabs demonstrated the persistence of case-specific POI DNA from epithelial cells up to 48 h, and the majority of positive samples were within 24 h. The authors also found that POI-derived epithelial cells were not detected in external genital swabs when collected beyond 12 h post assault.

In addition to DNA, the persistence of mRNA and miRNA in laundered or cleaned biological fluid stains has also been investigated. Mayes et al. [138] showed that mRNA and miRNA markers were able to detect blood and semen from stains exposed to various laundering conditions. They also tested blood and semen stains exposed to various environmental conditions relating to temperature, humidity and light for six months, and found that mRNA targets were observed through six months for some conditions but undetectable after 30 days for other conditions, whereas their miRNA targets persisted under all test conditions for the duration of the study. Furthermore, when investigating the level of blood-derived DNA and miRNA inside firearm barrels after cleaning with DNA-ExitusPlus™ (AppliChem GmbH, Darmstadt, Germany), Schyma et al. [139] found that while the DNA had been removed, the blood-specific miRNA was still detectable.

### 3.5. Prevalence and Background

This section summarises the ten papers identified that investigated the prevalence of DNA and presence of background DNA (Figure 2), plus one additional study that also provided comment on this subject matter but was primarily categorised in another topic. The prevalence of DNA from drivers, passengers and other individuals on a wide range of locations within vehicles driven regularly by one individual has been explored in three studies [140,141,142]. These studies found that DNA was able to be collected from all targeted sites, and yields and profile compositions varied between sites, with relatively higher yields retrieved from steering wheels and seats. The authors found that the driver was always observed as a contributor in DNA profiles from sites on the driver’s side, in most instances being the sole or a major contributor, and the driver was also observed as a sole or a major contributor at several sites on the passenger side. They also found that DNA profiles of known recent passengers, close associates of the driver, and unknown individuals were observed on many of the sites on both the passenger and driver sides.

The prevalence of DNA from users in an office space was considered by Goray et al. [119]. They investigated the extent to which DNA, left by a temporary user of the office space that had been occupied by a regular user for an extended period, is detectable when the duration of their temporary occupancy and their general activities are known, as well as how readily the DNA of the regular user is still detectable after a known period of occupancy by another person. From samples collected from several items/surfaces within single use office spaces that had been used temporarily by another occupant for 2.5–7 h, the authors found that sufficient DNA for profiling was recovered from nearly all areas sampled. They found that the regular user of the office space was the major or majority contributor (as defined by the authors) to profiles from most items within the space, even after temporary use by another person. It was reported that detectability of the temporary occupier varied among offices and items tested, and whilst the temporary occupier was not observed on all items they touched, in most instances when detected, it was known that they had touched the surface at some stage. The authors also observed profiles from other individuals, including colleagues, family members and unknowns, that were present on several items, mostly as minor contributors.

The study by Szkuta et al. [143] considered background levels of DNA on common entry points to homes (rarely contacted external and internal windowsills and edges, and frequently contacted entrance door handles) occupied by known individuals with some general knowledge on the history of use. They found that interpretable DNA profiles were able to be generated from nearly all door handles (all mixed profiles) and internal windowsills and edges (mostly mixed profiles), but the majority of external windowsills and edges yielded limited to no DNA profile information. The authors reported that the individuals living in the house tended to be main contributors to the profiles, and that the last person to make contact with a door handle was observed as a contributor in most but not all profiles. The authors also observed unknown contributors in the profiles of many of the door handle, internal windowsill and edge samples, and in most situations, but not all, they were a minor contributor.

The study by Albani et al. [144] investigated the extent of male-derived DNA presence on unused feminine sanitary products (tampons, pads, liners). The authors postulated, but did not test, that if such DNA was present, it could possibly be transferred onto the wearer and subsequently be detected in samples taken from intimate areas during a medical examination. However, the authors found no to very little background DNA present, with only 4 of the 52 items (1 tampon and 3 liners) exhibiting one or two Y-STR alleles and 1 additional item (pad) exhibiting two alleles using an autosomal STR kit, with all alleles appearing with low RFU signals.

To elucidate the level and origins of background DNA on worn upper garments, Szkuta et al. [117] and Szkuta et al. [118] collected samples from several areas (two internal and six external) of two garments (both owned and previously worn by the participant) each worn on separate occasions, for 6 to 15 h on a work day and a non-work day, after being washed. They also recorded details of the history of the garment, and the interactions and activities during wearing, and collected samples for reference DNA profiles from participants and their close associates. They found variation in the DNA quantity, profile composition and inclusion/exclusion of the wearer and their associates, among participants, garments worn on different occasions and the garment areas sampled. The authors reported that DNA from the wearer of a garment was found on all internal and external areas sampled, with the best quality profiles corresponding to the wearer obtained from internal collars followed by internal cuffs. They also found that DNA from individuals living or sharing a space with the wearer was frequently detected in samples from external areas of the garment. In such samples, the contribution of the wearer to the DNA profile was occasionally absent or minor relative to the presence of the close associate; this was most frequently observed in samples taken from the external back of the garment.

A further study by Murphy et al. [145] examined the background levels of male DNA on the inside front of clean, but not new, pairs of underpants worn for a day by a female who was asked to abstain from sexual intercourse for 48 h prior to taking part in the study. The authors reported that, of the 103 samples taken after a day of wearing, 83 showed the presence of male DNA to varying quantities (as detected by the Quantifiler Trio DNA quantification kit (Thermo Fisher Scientific, Warrington, UK)) and 24 contained sufficient DNA to proceed to profiling. Of those 24 profiled samples, 12 samples showed the detection of the Y chromosome and varying numbers of minor alleles (not from the female wearer) using the autosomal NGM SElect kit (Thermo Fisher Scientific, Warrington, UK), 5 gave a complete Y-STR profile, and 18 gave incomplete Y-STR profiles. Each of the samples that gave a complete Y-STR profile was from a female who cohabited with a male partner and the profile matched that of the partner where a reference profile was available (four of five). Based on the results of their study, the authors concluded that the finding of a Y-filer profile on the inside front of underpants worn by a female is not expected in the absence of sexual contact. Further, in the absence of sexual contact, a Y-STR profile, if present, is more likely to be obtained from a cohabitating male rather than from a non-cohabitating male in social contact with a female.

Two studies included investigation of the impact of cleaning on levels and composition of background DNA on various surfaces. Reither et al. [146] found that domestic cleaning methods (e.g., vacuuming, mopping with cleaning agents that did not contain cholic acid) had limited impact on the quantity of DNA recovered from various types of flooring (e.g., carpet, tiles), but often altered the DNA profile composition of the samples collected when comparing the profile from the post-cleaned area with the profile from the corresponding pre-cleaned area. Similarly, in experiments to determine the impact of a range of cleaning agents and cleaning actions using DNA-free towel pieces and sponges on small blood stains, saliva stains and fingermarks on plastic table tops and fabric surfaces, Helmus et al. [147] found that the DNA was redistributed to other areas of the item by the cleaning action. They also found that the cleaning agent had little impact on the ability to generate DNA profiles of the sample collected, with the exception of chloric agents, which the authors asserted rendered almost everything DNA-free.

### 3.6. Manner of Handling

This section reports ten studies that investigated manner of handling (Figure 2). We have broken down this section into further subtopics to categorise the studies into elements of handling, including time, pressure, and testing of various scenarios. We have also included studies on non-contact DNA transfer in this section.

#### 3.6.1. Handling Time

In their study of the impact of handling time by a non-wearer of a worn brassiere, Sessa et al. [93] found that the contact duration does not appear to have an effect on the amount of DNA deposited and when a major contributor is detected on a garment. Outcomes of their experiments (which included small specific target areas of known contact by the ‘handler’) also showed that the person handling the garment last contributed the most to the recovered DNA, even though they may have touched the garment for only a few seconds.

#### 3.6.2. Pressure

Examining the effect of deposition pressure (using weights from 0.1 to 10 kg) applied by fingers on the quality of latent fingermarks and DNA STR profiles, Hefetz et al. [148] found that as deposition pressure increased, so did the length and width of fingermarks and the efficiency of DNA profiling from the fingermarks. The latter was based on increases in the amount of DNA recovered, number of amplified loci, and number of forensically useful DNA profiles, which were observed for each of the substrates tested (glass, polythene, paper).

#### 3.6.3. Scenario Testing

The transfer of DNA to clothing under a number of case-relevant scenarios was investigated by Szkuta et al. [118] and De Wolff et al. [149]. Szkuta et al. [118] examined whether an ‘activity partner’ was discernible on clothing worn by another individual after specific interactions: ‘contact’ where the final action during the wearing of the garment was an embrace with a known other individual; ‘close proximity’ where the final action during the wearing of the garment was to travel to and from a nearby café to have a meal with a known other individual; ‘physical absence’ where the wearer of the garment spent most of a working day in an office of a known other individual, in the absence of the other individual. The study did not find any DNA from the activity partner on the garments after the wearer had gone on an outing, even though they had been in close proximity with each other for a while. However, the study did observe DNA from the activity partner on several areas of the garments following the embrace and after temporarily occupying another person’s office. It was reported that particular areas of the garment were more prone to acquiring DNA from the hugging partner or the office owner than others, and their detection as a major or minor component was deemed activity dependent. When investigating DNA transfer to clothing within vehicles, De Wolff et al. [149] found that in many instances, DNA from a regular driver of a vehicle could be recovered from samples collected from the external back of the upper garment, and from the seat of the trousers pants, worn by an incidental driver (30 min) of that vehicle soon after departing the vehicle. This highlighted a potential avenue of indirect transfer that may be relevant during investigations of particular types of offences.

DNA transfer within specific scenarios that can occur during sexual assaults was investigated by Ramos et al. [150]. Their study recorded where underwear was contacted and how much DNA was transferred by volunteer ‘offenders’ during six types of mock sexual assault scenarios: removing underwear; removing brassiere; digital penetration of the vagina from the front; grabbing breasts over the top of the brassiere; digital penetration of the vagina from the rear; grabbing the breasts under the brassiere from the rear. The authors found that the pattern of contact varied for different activities and identified the areas of underwear most commonly contacted from various sexual assault activities. They demonstrated that some areas corresponded to areas typically sampled during casework and others less so, thus providing information to help improve sample targeting. They too observed several instances of a colleague or cohabitating partner of mock offenders being detected within a DNA sample.

A study by Gosch et al. [151] considered trace DNA characteristics (including DNA yield, number of contributors, relative profile contribution for known and unknown contributors, LRs) of samples recovered from various surfaces of two types of firearms handled in four realistic casework-relevant scenarios. In each scenario, the firearm had background DNA deposited over four days by the mock owner. The four different activities then undertaken with the firearms included: the mock owner performed a shooting scenario; a second individual performed the same shooting scenario as the mock owner; a second individual imitated a longer and more intensive contact with the firearm; a second individual performed the same shooting scenario as the mock owner, then wiped the firearm using a dry cotton towel for 15 s simulating fingerprint removal. The authors observed a large variability of trace characteristics that was partly attributable to the way the firearms were handled, the firearm surface types sampled, and factors of inter- as well as intra-individual variability.

When considering DNA transfer to knife handles, particularly within opportunistic crimes, Butcher et al. [152] conducted experiments to generate data to help address consideration of questions relating to whether the DNA obtained comes from the regular user, the last user (ostensibly, the user at the time of the crime) or from indirect transfer events. The authors found that regularly used knives that were then used for just 2–60 s by a second person, performing a stabbing action, resulted for three of the four participant pairings in a decrease in contribution from the regular user’s DNA with simultaneous increase in contribution from the second user’s DNA. They reported that the trend appeared to be due to a decrease in regular user DNA via transfer to the second user’s hands, rather than an increase in DNA deposited from the second user, but a deviation from this general trend was observed for the fourth participant pairing. While they found a low level contribution of the non-handler’s DNA on most knife handles, one volunteer deposited a similar amount of DNA through regular use as the amount of indirectly transferred unknown DNA deposited by another volunteer’s hands, leading the authors to indicate that caution should be taken when relying solely on absolute quantities of DNA to inform evaluative interpretations and that other parameters, such as profile quality and relative contributions to mixed profiles, should also be taken into account. However, when also considering DNA transfer within stabbing scenarios, Samie et al. [153] observed that the quantity of DNA generally reduced when moving from hand (the quantity collected when sampling directly from a hand) to primary transfer (the quantity collected from a surface handled by a hand) and, subsequently, to secondary transfer.

To assist activity level evaluation, Euteneuer et al. [154] investigated the correlation between shooting distance and the amount of blood backspatter in/on a used firearm. The authors could not establish any meaningful correlation between backspatter distribution and DNA yields, the shooting distance and the condition of the wound channel. 

#### 3.6.4. Non-Contact Transfer

The first study to consider non-contact transfer with dried biological materials was published by Thornbury et al. [155]. They investigated the possibility of indirect DNA transfer without contact from various substrates (two different non-porous hard substrates and two different porous, soft substrates) after various biological materials (blood, saliva, semen, vaginal fluid, touch DNA) deposited on them had dried and after applying two types of agitation (tapping—all substrates; stretching—all fabric substrates). The authors found that detectable levels of indirect transfer of DNA without contact were possible. They also demonstrated that the type of biological material and substrate affected the drying time and characteristics of deposits, and that the rate and percentage transfer appeared to be dependent on the type of agitation, substrate, biological material and its drying properties.

### 3.7. Shedder Status

This section summarises the one paper where shedder status was deemed the most relevant DNA-TPPR aspect (Figure 2), plus seven additional studies that also provided comment on this subject matter but were primarily categorised in other topics. When investigating shedder status as a possible factor influencing the extent of secondary DNA transfer to a crime scene when the offender wears working gloves, Otten et al. [156] initially determined the shedder status of a group of individuals, finding that good shedders deposited higher amounts and quality of DNA onto objects compared to bad shedders. The authors then conducted experiments where participants were paired into groups of various combinations of good and bad shedders, with the first participant of each pair wearing working gloves to pack and carry boxes, and then, two days later, the same gloves were worn by the second participant to break-in using a screwdriver. The study reported that good shedders, overall, deposited more DNA than bad shedders, both onto the outside and inside of the gloves regardless of being the first or second wearer and user of the gloves. The authors also found that a DNA profile of the first wearer was found on the screwdriver in several instances, and in each of the situations where this was observed, the first user had been a designated good shedder, confirming the possibility of a person’s DNA profile being found on an object they never handled.

Several other papers demonstrated or alluded to observing differences in the quantities of DNA deposited, which was attributed to differences in shedder status, for example [51,78,150,152]. However, Miller et al. [95] found that individuals who deposited extremely large amounts of DNA by touch one day may deposit no detectable DNA on another day despite a restriction on handwashing close in time to deposition, leading the authors to question the concept that there are consistently good shedders, at least through the palms of hands. Similarly, Samie et al. [153] noted that while a person could be judged as a good shedder overall, when considering a single experiment, that same person could be a very poor shedder. They suggest that when assessing the probability of observing a given quantity of DNA, for a given donor, the whole distribution should be accounted for and not only its mean value. The authors also showed that the ‘transfer proportion’ (quantity of DNA transferred onto a surface over the initial quantity on the hand) may vary between participants and will depend on the type of transfer (primary versus secondary), indicating that the quantification of DNA on one hand cannot be used to infer the shedder status and asses what will be transferred onto a surface.

Like shedder status determination from finger/hand marks, a study by Kanokwongnuwut et al. [42] found that the amounts of cellular material deposited by lip-prints is consistent for individuals, but different among individuals. The authors were able to categorise lip shedder status from heavy to light, but they did not observe a correlation between lip-print shedder status and the shedder status of the individual determined from thumb prints.

### 3.8. Contamination

The potential unwanted transfer of DNA during a forensic examination (or contamination) can also be relevant and has been considered in four of the studies identified (Figure 2). Three of these studies looked at the potential of DNA contamination via items used during an examination in the laboratory, specifically gloves and fingerprint brushes [157,158,159]. In the study by Goray et al. [157], a number of laboratory-based evidence recovery personnel were videoed performing a range of examinations and several factors were assessed to evaluate the risks of DNA loss, addition and redistribution via gloves. The factors included: what each glove touched and in what sequence; the number, duration and type of contact made by each glove with the item under examination, tools used and other surfaces; when gloves were replaced; DNA profiles of samples taken from worn gloves at the time of replacement against those relating to the item under investigation, the examiner, and other staff members. The authors observed that many different surface areas are touched by gloves during examinations, and differences exist among examiners in what they touch and when they change gloves. They reported that, in many instances, the case-associated person of interest was observed within the profile generated from DNA retrieved from outer surfaces of most gloves sampled at time of replacement. They also observed DNA profiles of the examiner, or other staff members, predominantly in samples taken from the first and last gloves used during the examination, which were associated with removing the exhibit from the packaging and repackaging it. Their evaluation deemed that several of the observed contacts made by the gloves were a high contamination risk event. Furthermore, Van den Berge et al. [158] performed tests showing that DNA from hands transferred to various parts of the external side of laboratory gloves while being donned. With respect to used fingerprint brushes, the study by Nontiapirom et al. [159] produced results verifying findings that human DNA can persist on fingerprint brushes after use, such that fingerprint brushes can be a potential contamination risk when reused.

Two of the identified studies also considered the effectiveness of contamination-minimisation procedures within the laboratory [158,160]. In the above study by Van den Berge et al. [158], they also performed tests providing outcomes supporting the use of 0.3% sodium hypochlorite solution or commercially available RNase AWAY™ (Thermo Fisher Scientific, Waltham, MA, USA) as decontamination reagents to clean and subsequently dry the exterior of donned gloves prior to entering the laboratory and/or handling items of evidence. The authors reported that the results precipitated an update to their laboratory’s contamination risk reduction strategy. The study by Basset and Castella [160] compared the number of DNA contamination events by laboratory staff before and after the implementation of additional contamination minimisation procedures (that included: physical separation between living environments (e.g., offices, cafeteria) and laboratory zones; personal objects prohibited from laboratory zones; double gloving; use of different disposable lab coats within different laboratory rooms; lab coats changed daily; stain collection split among different operators so that the person in contact with the swab does not touch other surfaces; automation of DNA extraction for standard traces) and found that the number of such contamination events declined by more than 70% after implementation of the procedures.

### 3.9. Microbiomes

Fourteen studies (Figure 2) delve into the opportunities to identify individuals, or associate them with a location or an activity, that may be offered from analysing microbiomes in the absence of and/or in addition to traditional DNA evidence. These include investigations into:

- Classification of individuals and the potential to detect sexual contact using the microbiome of the pubic region [161].
- Bacterial communities associated with cell phones and shoes of the same person [162].
- Microbial communities of a wide range of known body sites and the detection of the source of different microbial contributions in mixtures of different body sites or with soil [163].
- Distinguishing microbiome communities of saliva, skin and mixtures of both as a potential tool for identifying samples of single and mixed biological materials [164].
- Correlations between the presence of certain bacterial species on a donor’s hands and personal characteristics [165].
- The direct and indirect transfer of microbiomes between individuals [166].
- The ability of several commercial kits to recover sufficient bacterial DNA from fingerprints to generate a microbiome profile [167].
- Generating both STR profiles and microbial population profiles from the same DNA extract of each sample to evaluate the sub-source and source questions, respectively in two forensic cases [168].
- The usefulness of the likelihood ratio approach for the evaluation of source attribution in microbial forensic cases to facilitate the interpretation of evidence in legal proceedings [169].

In addition, Young and Linacre [170] provided a review of studies on the potential and limitations of utilising microbiome analyses in forensic science, especially in relation to skin microbiome. Meanwhile, Jurkevitch and Pasternal [171] provided a review on the use of soil bacterial community comparisons to assist investigation of alleged crimes. Each of these studies and reviews, as well as a review by Neckovic et al. [172], indicated some positive results and potential useful applications, but also highlighted a range of limitations of their applicability and the challenges of microbial profiling for forensic science casework applications. These challenges are further illustrated by investigations of the presence of microbiomes within forensic evidence recovery laboratories, and the potential for microbiomes to transfer during examinations [173,174].

### 3.10. Evaluating DNA Evidence

This final section of the synopsis reports papers that consider the evaluation of DNA evidence given propositions at different levels within the Hierarchy of Propositions. Ten papers with a primary focus on this subject matter are included (Figure 2), along with one other that was primarily included in another topic, and are reported within two subsections: setting propositions and use of Bayesian Networks, and other activity level assessment-related matters.

#### 3.10.1. Setting Propositions and Use of Bayesian Networks

When evaluating the value of biological evidence, it is important that the propositions are formulated appropriately. Gill et al. [120] presented guidelines from the DNA Commission of the International Society for Forensic Genetics (ISFG) on determining and formulating the propositions for the evaluation of biological traces, given activity level propositions. This paper included eleven specific recommendations and provided examples to illustrate the principles espoused. The authors also noted that suitable propositions are ideally set before knowledge of the trace DNA evidence, that data relevant to the case in question be gathered to assign probabilities, and that Bayesian Networks (BNs) are useful to help consider all relevant possibilities. Taylor et al. [175] also stressed the importance of properly constructing the propositions. The authors indicated the need for a clear summary of propositions representing the views of the prosecution and defence, accompanied with details on the agreed and contested information and assumptions by the scientist, to assist in carrying out an evaluation and in understanding by the courts. Furthermore, Buckleton et al. [176] showed an example where considerations of DNA-TPPR are required to inform sub-source level proposition setting, specifically to condition the calculation on the presence of DNA of an assumed contributor. The authors showed this in relation to the number of contributors, as this is likely to impact on the LR at the sub-source level. 

Several studies focussed on the use of BNs to evaluate DNA results given competing propositions, including Taylor et al. [177], Gill et al. [120], Volgin [178], De March and Tarino [179], and Samie et al. [180]. Examples of the use of BNs to evaluate biological traces given activity level propositions are provided in: the supplementary material within the article of Gill et al. [120] (that is accessible in the online version); and by Volgin [178] involving the use of a T-shirt as a balaclava. Further, De March and Tarino [179] provided an insight into the application of BNs to real criminal cases through the assessment of the probative value of different analyses related to some biological material retrieved from an object of interest. 

Two studies went further to consider the applicability of BNs to the complexities of issues of DNA transfer. Taylor et al. [177] showed the value of an Object-Oriented Bayesian Network (OOBN) to tackle cases involving repeating elements of complex and various DNA transfer mechanisms and indicated that, as new data on DNA-TPPR factors become available, these can be readily accommodated by the general overall OOBN architecture. Further, Samie et al. [180] showed that BNs can handle the complexity of variables associated with DNA-TPPR to inform activity level assessments. Their study demonstrated the uses of BNs and simulation methods to identify the variables impacting the value of evidence assessed under activity level propositions. The authors did so by focussing on trace DNA evidence recovered from knife handles allegedly involved in stabbing instances, showing that some variables had greater bearing on the variation of the LR than others. In the situation they investigated, the authors reported that the identified key variables were associated with the sampling, the extraction efficiency, the background and the quantity of DNA on hands, and they asserted that data acquisition should focus mainly on those variables with the greatest impact.

#### 3.10.2. Other Activity Level Assessment-Related Matters

In his commentary, Gill [181] pointed out that the increased sensitivity of modern DNA profiling techniques means that DNA may be recovered that is irrelevant to the crime and stressed the importance of scientists, lawyers and judges to be aware of the limitations of DNA profiling evidence. This was echoed by Sessa et al. [93], who also pointed out the need for everyone in the medico-legal community to know the power of touch DNA and understand the potential limitations associated with it. Gill [181] went on to note that the presence of DNA does not tell us when or how it got there, so additional context is needed from other non-DNA evidence, which if ignored could result in miscarriages of justice. He provided several real case examples to highlight how ignorance of key aspects, such as background DNA, secondary transfer, and laboratory contamination, can influence case outcomes. Similarly, Perepechina [182] referred to a retrospective analysis of a case where consideration of transfer issues was pertinent. Given these issues, he [181] also pointed out the need to educate stakeholders, including all parties engaged in the criminal justice system, with respect to the various facets relating to activity level assessments.

An additional concern highlighted by Gill [181] is the occurrence of carryover of the strength of evidence associated with a DNA ‘match’ to weight of evidence associated with activity level propositions. He reported that this cannot be done; activity level assessments are to be undertaken separately using different information and generating a different statistic. This point is also made by the DNA Commission of the ISFG [120].

Apart from the few calls for specific further research noted above, many of the other publications incorporated in this synopsis, in their discussion sections, also incorporate suggestions/recommendations/advocacy for further research to enlighten our understandings and advance the acquisition of relevant data to improve our abilities to provide activity level assessments. From challenges encountered in their study, Dunhill and Chapman [183] encouraged authors to publish results of studies on indirect transfer of DNA in a more streamlined fashion to allow for systematic analysis of data across studies. Further, De March and Tarino [179] indicated that while the data available to inform probabilities are limited and extrapolation from published data for practical use in a case analysis can be troublesome, this should not prevent the forensic scientist from providing a statement at the activity level when the circumstances of a case require it. 

## 4. Reflections on the Research Efforts to Meet the Challenges

Based on the focus, scope and depth of the research activities published over the last few years, we reflect that overall, some progress has been made to meet the many challenges, but much of the challenges noted in the introduction remain and require more effort to address them. While several of the recent studies have provided means of improving the detection and recovery of DNA and DNA profiles of relevance from crime-related items, more effort will be required to determine the best practices given the specific circumstances encountered. Of the 144 articles included in the synopsis, relatively few (35) primarily focus on aspects of persistence, DNA prevalence and background DNA, manner of handling and shedder status (Figure 2). Of these, few provide the highly desired data to help assign probabilities of obtaining profile types given particular sets of circumstances, especially those that are relatively commonly encountered. The latter is partly due to the relatively recent and increasing need for such data, and the requirement for sufficient repeats to be conducted to generate robust frequency data, which may place this type of research beyond the scope of some research groups. The multitude of situations and associated variables for which probabilities are desired provides scope for conducting many future research projects to provide welcomed outputs.

Some additional research efforts have been directed at determining and mitigating risks of contamination of evidence (see Section 3.8). Further research may highlight additional risk factors and means of mitigating these, thus providing opportunities to enhance the integrity of the evidence throughout the forensic process and the interpretation thereof.

Some research has been conducted on other elements of information that may be gleaned from deposits of biological materials to facilitate activity level evaluations (see Section 3.1, Section 3.3, Section 3.9). Further studies towards improving abilities to accurately determine the TSD of various types of biological material in various circumstances (preferably even of different components within mixed samples), and into microbiome profiles that inform on the environment that a sample is associated with, and/or its source, and/or provide an avenue for identity determination in the absence of human DNA, may assist future activity level evaluations.

To assist with activity level evaluations, BNs are starting to be utilised more (see Section 3.10.1), such that any developments towards making BN-related software packages even more user friendly for forensic practitioners may further facilitate their utilisation and the standard of the guidance provided to the court. However, there appears to be little recent human factors-related research effort directed towards facilitating the development of competencies of those tasked with providing activity level-related guidance and how to best convey this guidance to other stakeholders. Such research, as well as human factors research relating to individuals and processes associated with the collection of evidence and maintaining its integrity, would be welcomed as they may identify improvement opportunities in the means of training experts and the quality of tasks undertaken and the services provided.

Encouragingly, very recent publications (i.e., articles published after conducting the systematic search, or during the search period but not captured) demonstrate the realisation of the need for, and the efforts supporting, ongoing research activity towards meeting the broad range of outstanding challenges. Examples of such publications of studies addressing identified knowledge and data gaps include:

- DNA profile success of samples from casework-related items [24].
- Impacts of surface roughness and physicochemical interactions on deposits of biological materials [184].
- Impacts of chewing gum on drying properties of saliva [185].
- Prevalence and persistence of saliva in vehicles [186].
- Exploring means of determining an individual’s shedder status.
- Consideration of distance and time with respect to accumulation of DNA within offices on surfaces that are not touched [187].
- Evaluation of profiles retrieved from the outside of gloves after simulating transfer scenarios related to breaking and entering [188].
- Findings indicating that cities around the globe have distinct microbial taxonomic signatures [189].
- Development of an LR framework incorporating sensitivity analysis to model multiple direct and secondary transfer events on skin surfaces [190].
- Highlighting the importance/value of considering common sources of unknown DNA [191].
- Assigning weight of cell type testing results [192] when evaluating findings given activity level propositions.

These studies demonstrate the realisation of the need for, and the efforts supporting, ongoing research activity towards meeting the broad range of outstanding challenges. 

## 5. Industry Actions towards Addressing Challenges

### 5.1. Research

Many of the reviews and research papers referenced in the previous sections note the need for more research to better inform our understanding of variables affecting DNA-TPPR and supplying relevant data to determine probabilities given a set of circumstances. Meakin et al. [193], apart from also advocating this, urged for better designed research studies to cater for this need. In particular, they expanded on the recommendation of Gosch and Courts [36] for different experimental designs to be employed when generating probabilities for activity level evaluations as opposed to investigating DNA transfer mechanisms. They recommended that the former requires iterations of realistic experiments (e.g., a casework-relevant scenario performed once by multiple participants), whereas the latter require repetitions of highly controlled and potentially unrealistic experiments (e.g., a single participant performing the same action multiple times). Meakin et al. [193] also directed readers to the ISFG recommendations [120], which provide further advice for designing experiments to generate activity level probabilities.

Evident from the authorships and their affiliations, forensic laboratories, alone or in collaboration with other forensic laboratories and/or academic institutions, are major contributors to much of the published DNA-TPPR-related research. Much of this is being funded by internal resources. Major research funding bodies are also increasingly supporting DNA-TPPR-related research. For example, the National Institute of Justice (NIJ) in the United States of America has funded over 60 DNA-related projects to the value in excess of USD 42 million during 2018–2020 [194,195], which include a wide range of DNA-TPPR-related research projects, and the European Network of Forensic Institutes (ENSFI) has recently funded a large multi-institutional collaborative project to generate accessible DNA transfer rate data [196].

### 5.2. Education and Training

Apart from the need for research to improve our understanding and generate data to allow the assignment of probabilities given a set of circumstances, there are other challenges to be met by relevant industry stakeholders. These include aspects relating to the education and training of those tasked with providing expert evidence on DNA-TPPR-related matters, building of awareness of aspects associated with DNA-TPPR among crime scene attendees and those within the legal fraternity, availability and utility of published data, and setting of standards associated with DNA-related activity level evaluative reporting. Steps are being taken to address these challenges.

DNA-TPPR-related research findings, including unpublished findings, are being presented and discussed within dedicated sessions and workshops at international forensic conferences attended by forensic, policing and legal practitioners as well as academia. For example, Congresses of the International Society of Forensic Genetics [197]; Gordon Research Conferences: Forensic Analysis of Human DNA [198]; American Academy of Forensic Sciences (AAFS) conferences [199]; the International Symposium on Human Identification ((ISHI) [200]; and the symposium on Recent Progress on Forensic Sciences and DNA Transfer organised by Laboratoire d’Hématologie Médico-Légale [201].

Further education/upskilling of stakeholders on the topic of DNA-TPPR and the associated activity level evaluative reporting is also being made possible through the development and availability of dedicated programs such as summer schools on various aspects of forensic genetics organised by the International Society of Forensic Genetics [202] and university-based courses such as the Certificate of Advanced Studies on ‘Statistics and the evaluation of forensic evidence’ run by the University of Lausanne [203]. Freely accessible courses and webinars have also been produced, such as that on ‘Challenging Forensic Science: How Science Should Speak to Court‘ made available by the University of Lausanne [204] and the series of webinars on DNA mixture interpretation and probabilistic genotyping organised by the Federal Bureau of Investigation (FBI) Laboratory and NIJ’s Forensic Technology Center of Excellence [205].

The skill set required to become expert at the provision of guidance on DNA-TPPR at the activity level is distinct from those required to provide guidance at the DNA sub-source level, biological material source level or non-DNA activity level. Recognition of this can be an important first step for laboratories to help define the necessary enrichments and changes to a suite of methodologies, protocols, training units and authorisations. Introduction of these is then to be managed, such that the increasing demands for such services can be met at the expected standards. Most forensic DNA experts trained over the last twenty years have been trained to provide statements at the sub-source level. A forensic expert solely trained and competent at providing DNA evidence at the sub-source level should not provide opinion at the activity level without also being trained and authorised to do so.

An example of a jurisdiction with a competency testing program specific to evaluation of forensic biology findings given activity level propositions can be found in the Netherlands [206]. Experts can submit an application for registration as an expert in the field to the Netherlands Register of Court Experts (www.nrgd.nl). They are subsequently assessed by a committee of both scientific experts and legal professionals against a standard that has been developed by a group of international experts in the field. The standards demarcate the field of ‘DNA activity’ from ‘DNA source’ and ‘DNA kinship’ and list the minimum requirements for experts in these fields. These requirements cover training, experience, methodology, reporting, and knowledge of the Dutch legal system [207]. Registered individuals must demonstrate their ongoing competency to the Board on a regular (five year) basis.

### 5.3. Availability and Sharing of Data

Kokshoorn et al. [208] and Gosch and Courts [36] advocated the need for sharing of research data associated with DNA-TPPR. They also indicated the type of information that is relevant when interpreting the generated data and should be made available when publishing DNA-TPPR-related research (or making it available on sharing platforms). These included information on: item (type of item, construction and surface composition, etc.); item history (handling, packaging, etc.); sampling (surface area targeted, sampling method, etc.); analysis (DNA extraction, amplification, CE, etc.); and interpretation (allele calling, statistical model, parameter settings, proposition setting, NoC determination, etc.). Kokshoorn et al. [208] also advocated for harmonisation and standardisation in how some of this information should be recorded, to further assist utilisation of the data. Gosch and Courts [36] constructed a database for entry and accessibility of such data, added the data from relevant publications and requested researchers to submit the relevant data to DNA-TrAC [37]. DNA-TrAC contains 311 entries of relevant research articles published in the period 1993–2017. Detailed information is provided per publication covering a wide range of aspects relevant to understanding DNA-TPPR and utilising data for activity level evaluations. It also provides the ability to sort information based on various parameters including general details of the publications, questions being addressed by the studies, transfer scenarios in the context of a specific type of activity, categories of study object (e.g., primary deposit, further transfer, background DNA, persistence, recovery), and variables affecting DNA-TPPR. The website also includes a list of 17 review articles published in the period 2002–2020 with a summary of the topics addressed by each as well as a file listing 33 articles relevant to DNA-TPPR published during the period 2018–2020 for which the details have not yet been entered into the DNA-TrAC database. The last version fully and actively curated by the Gosch and Courts was uploaded in December 2019 and a request is issued for others to assist with the maintenance of the database. DNA-TrAC is a useful portal to source DNA-TPPR information and data that may be relevant to a range of stakeholders. Maintenance and upkeep of this database, or the creation of something similar, would be an asset to the forensic community and thus desirable.

A separate relevant database (‘BDATT-TTADB’), constructed with funding support from research grants from the Social Sciences and Humanities Research Council of Canada, has recently become available [209,210]. The database was built with the aim of being relevant for practitioners to interpret transfer traces at activity level, integrating transfer experiments in an operational situation [209]. It provides information for a wide range of trace types, including DNA and biological fluids. It allows for easy searching according to type of trace, type of study (including transfer, persistence, recovery, background, contamination), key words (as determined by the database custodians, not the ones listed in the associated publication) as well as aspects such as authors. Apart from the general information regarding the publication (i.e., title, authors, journal), it provides a copy of the published abstract, a summary statement regarding the studies’ experimental conditions and results, a summary statement regarding the relevancy of the study for the Canadian environment, and a link to the publication. The database includes several hundred papers relating to DNA-TPPR and is updated regularly [209,210]. This database resource provides an excellent means for any interested parties to identify potentially relevant studies. However, those seeking some of the finer details often required when conducting activity level evaluations, as described above, will need to examine the identified literature more closely. An additional source of relevant data will also be made available in early 2022, when the UK Body Fluid Forum launches a digital collection of casework-informed research and case reports to facilitate sharing of experiences and data specifically relevant to informing evaluation of biological findings given activity level propositions. When available, these articles will be accessible here: https://www.sciencedirect.com/journal/science-and-justice/special-issues. Further development and maintenance of databases containing all the relevant information that allow easy mining of desired finer details, and further efforts towards harmonisation and standardisation of the data generated and shared as suggested by Kokshoorn et al. [208], would be welcomed.

### 5.4. Standards and Guidance

Many standards and guidance on how to conduct DNA-related activity level evaluative reporting are being provided by governing bodies. For example, the Forensic Science Regulator in England and Wales [211] and the DNA Commission of the ISFG [120,212]; there is also an ISO standard relating to forensic science reporting currently under development [213]. Additionally, some of the fallacies associated with the reporting of DNA-TPPR matters, that forensic practitioners need be cognisant of and avoid when providing guidance to the triers of fact, are highlighted by Meakin et al. [193]. 

Forensic laboratories are also investing in developing in-house methods, procedures and training modules to facilitate the conduct of DNA-related activity level evaluations and the training of their staff to do so. However, unlike many other forensic disciplines, independent ongoing proficiency testing in all the relevant elements associated with DNA-related activity level evaluations for those authorised to provide expert opinion to assist triers of fact is not readily available. Creation of a good fit for purpose internationally available proficiency testing program would facilitate the provision of high-quality guidance by forensic practitioners.

The National Institute of Standards and Technology (NIST) in the USA has very recently (June 2021) published a draft of a scientific foundation review in relation to the interpretation of DNA mixtures [214]. It also contemplates several of the points we raised earlier. It is a wide ranging comprehensive in-depth review that provides an overview of the past, present and future of DNA mixture interpretation and identifies challenges and pathways to deal with them. It includes a general introduction; principles and practices relating to DNA mixture interpretation; sources of data and information; delving into the issues pertaining to the reliability of DNA mixture measurements and interpretation as well as the context and relevance of DNA mixture interpretation (with details on various factors associated with DNA transfer); consideration of the potential and limitations of new technologies to provide solutions to existing problems. The chapters and appendices are accompanied with highlighted key takeaways, principal points and/or future considerations. Takeaways that directly relate to DNA transfer and activity level reporting, presented in chapter 5 of the review [214], include:

- #5.1: DNA can be transferred from one surface or person to another, and this can potentially happen multiple times. Therefore, the DNA present on an evidence item may be unrelated (irrelevant) to the crime being investigated.
- #5.2: Highly sensitive DNA methods increase the likelihood of detecting irrelevant DNA. When assessing evidence that involves very small quantities of DNA, it is especially important to consider relevance.
- #5.3: Highly sensitive methods increase the likelihood of detecting contaminating DNA that might affect an investigation. Contamination avoidance procedures should be robust both at the crime scene and in the laboratory.
- #5.4: DNA statistical results such as a sub-source likelihood ratio do not provide information about how or when DNA was transferred, or whether it is relevant to a case. Therefore, using the likelihood ratio as a standalone number without context can be misleading.
- #5.5: The fact that DNA transfers easily between objects does not negate the value of DNA evidence. However, the value of DNA evidence depends on the circumstances of the case.
- #5.6: There is a growing body of knowledge about DNA transfer and persistence, but significant knowledge gaps remain.

### 5.5. Cooperation with Other Scientific Disciplines, the Legal Community, and Police

The compartmentalised nature of the scientific disciplines in many modern forensic laboratories hampers the proper evaluation of forensic findings [215]. Interdisciplinary assessments of traces increase the impact of the forensic sciences on the criminal justice system (CJS), both improving efficiency by reducing unnecessary examinations and improving understanding of the combined weight of the findings given case-relevant propositions at the activity level [216]. 

Case-relevant information is structured in propositions, assumptions, and other, undisputed case information [175]. For any evaluation of findings given activity level propositions that a scientist is asked to perform, it is crucial that all parties in the CJS are in a position to share task-relevant information with the scientist. 

Education and training of stakeholders in the CJS is a crucial step to reduce common misconceptions of what evaluations given propositions at the activity level are, as well as to achieve the desired communication and collaboration between the scientist and the other actors in the CJS. Efforts are being made in several jurisdictions, for instance, by providing guidance documents for legal professionals [217,218,219,220]. 

## 6. Concluding Remarks

Much progress has been made recently towards addressing identified challenges concerning the provision of expert guidance in relation to DNA-TPPR-associated activity level evaluations sought to facilitate just outcomes during legal proceedings. However, many challenges remain. These include in relation to:

- Quantity and quality of research directed at informing our understanding of DNA-TPPR and building the data needed to assign probabilities to DNA quantities and profile types being obtained given a vast range of circumstances.
- Harmonisation, standardisation, and accessibility of available data.
- Development of validated standardised methodologies and protocols to perform DNA-related activity level evaluations.
- Training and authorisation of experts to perform such evaluations and provide the guidance when needed as well as recognition of this expertise as a separate discipline.
- Education and cooperation of stakeholders within the legal fraternity.

Continued investment in addressing these challenges will improve the value and understanding of the data and the guidance given to the triers of fact.
